# Efficacy of Jinhua Qinggan Granules Combined With Western Medicine in the Treatment of Confirmed and Suspected COVID-19: A Randomized Controlled Trial

**DOI:** 10.3389/fmed.2021.728055

**Published:** 2021-10-12

**Authors:** XueDong An, Xi Xu, MingZhong Xiao, XiaoJun Min, Yi Lyu, JiaXing Tian, Jia Ke, SuPing Lang, Qing Zhang, An Fan, BinBin Liu, Ying Zhang, YaLing Hu, YaNa Zhou, JiaKai Shao, XiaoDong Li, FengMei Lian, XiaoLin Tong

**Affiliations:** ^1^Guang'anmen Hospital, China Academy of Chinese Medical Sciences, Beijing, China; ^2^Hubei Provincial Hospital of Traditional Chinese Medicine, Wuhan, China; ^3^GCP ClinPlus Co., Ltd., Beijing, China; ^4^Beijing University of Traditional Chinese Medicine, Beijing, China; ^5^Changchun University of Traditional Chinese Medicine, Changchun, China

**Keywords:** Jinhua Qinggan granules, COVID-19, randomized controlled trial, Chinese medicinal, treatment

## Abstract

**Objective:** To conduct a randomized controlled clinical trial to evaluate the clinical efficacy and prognostic value of Jinhua Qinggan granules in patients with confirmed and suspected coronavirus disease 2019 (COVID-19).

**Methods:** A total of 123 suspected and confirmed COVID-19 patients participated in this clinical trial and were randomly divided into Jinhua and Western medicine groups. For 14 days, the Jinhua group was treated with Jinhua Qinggan granules and antiviral drugs, and the Western medicine group was treated with antiviral drugs alone. We collected information on clinical symptoms, disease aggravation rates, and negative conversion rates of nucleic acids in patients, and observed the effects of anti-infective drugs.

**Results:** There was no significant difference in symptom improvement rates between the two groups, both confirmed and suspected patients (*P* > 0.05). Both treatments relieved symptoms such as fever, fatigue, and diarrhea. However, the Jinhua treatment was superior in relieving fever and poor appetite. Anti-infective drug use rates were significantly lower in the Jinhua group than in the control group.

**Conclusion:** Jinhua Qinggan granules combined with Western medicine could relieve the clinical symptoms of fever and poor appetite in COVID-19 patients, reduce the use of antibiotics to a certain extent.

**Clinical Trial Registration:** The registration number at China Clinical Trial Registry is ChiCTR2000029601.

## Introduction

Novel coronavirus-infected pneumonia is defined as an acute respiratory infection caused by a novel coronavirus. The World Health Organization (WHO) has named coronavirus disease 2019 (COVID-19) and its causative pathogen severe acute respiratory syndrome coronavirus-2 (SARS-CoV-2) (1, 2). Since December 2019, there has been a COVID-19 epidemic in Wuhan, Hubei Province. As the epidemic spread, COVID-19 has occurred throughout China and in many countries worldwide, causing a pandemic. The pandemic has had a serious impact on global society and the economy. WHO strongly recommends tocilizumab, systemic corticosteroids for severe and critical patients with COVID-19^1^. A systematic review of 13,412 patients with COVID-19 enrolled in 38 RCTs showed that the treatment of tocilizumab was associated with a reduction in COVID-19 mortality, but did not alter the severity and length of stay of COVID-19 (3). Meanwhile, colchicine, monoclonal antibiotics and anticoagulants are also being considered for covid-19 patients^1^. Colchicine has been shown to help reduce inflammation in several inflammatory diseases. A systematic review of 5,778 patients enrolled in 8 RCTs showed that colchicine can improve the prognosis and mortality of patients with COVID-19. It is recommended that colchicine be used routinely in the treatment of patients with COVID-19. However, more RCTs are needed to confirm the results of this study (4).

Studies have shown that there is a high probability of negative nucleic acid detection in respiratory tract samples from suspected patients (5). Management of suspected cases is an important link in preventing and controlling the source of infection and cutting off the transmission routes of COVID-19. Prevention and treatment of suspected cases could effectively improve the prognosis of patients who go on to develop COVID-19 and could effectively protect and treat non-COVID-19 patients, which could greatly improve the efficiency and level of front-line prevention and control. Therefore, suspected cases were also the focus of this study (6, 7).

Jinhua Qinggan granules have been recommended as a medication in the Diagnosis and Treatment Protocol for COVID-19 in China, and have previously shown remarkable efficacy in the fight against influenza A H1N1 (8, 9). The medication relieves the symptoms of fever, cough, expectoration, and shortness of breath. This clinical trial evaluated the efficacy of Jinhua Qinggan granules in relieving clinical symptoms and improving prognosis in confirmed and suspected COVID-19 patients, with the goal of providing new clinical evidence for COVID-19 treatment options.

## Methods

This study adopted a randomized, controlled, and open-label design. The subjects selected for treatment were from isolation sites in the Hongshan and Wuchang districts of Wuhan City, Hubei Province. The research protocol was approved by the Ethics Committee of Hubei Provincial Hospital of TCM (HBZY2020-C01-01). Informed consent was obtained from all participants. This study was registered with the China Clinical Trial Registry under the registration number ChiCTR2000029601.

### Subjects

#### Diagnostic Criteria

The COVID-19 diagnostic criteria for suspected and confirmed cases were based on the Diagnosis and Treatment Protocol for COVID-19 (Trial Version 7) (10).

#### Inclusion and Exclusion Criteria and Groups

The inclusion criteria for enrollment in this study were as follows: suspected and confirmed cases who met the above-mentioned suspected COVID-19 diagnostic criteria and had mild and common symptoms; 18–80 years of age, gender-neutral; and provision of informed consent.

Patients excluded from the study on the basis of the following criteria: clear evidence of bacterial infection; severe primary diseases of the heart, kidney, lung, endocrine, blood, metabolism, and gastrointestinal tract that may affect the patients' participation in the trial or the outcome of the study, those with a family or personal history of mental illness, those with allergies or multiple drug allergies, and pregnant or lactating women.

Patients who met the following criteria were excluded: those who did not meet the diagnostic and inclusion criteria, those who withdrew themselves without any observation data, those who could not adhere to the treatment or withdrew during the study for some reason, and those who violated the trial protocol.

#### Study Medication and Intervention

Jinhua Qinggan granules (SFDA approval No.: Z20160001, Juxiechang (Beijing) Pharmaceuticals Co., Ltd., Specification: 5 g/bag, Batch No.: 20200113) are light brown to brown in color, slightly fragrant, and bitter in taste. They are composed of 12 TCMs: *honeysuckle, gypsum, honey ephedra, fried bitter almond, Scutellaria, Forsythia, Fritillaria, Anemarrhena, burdock seed, Artemisia annua, peppermint*, and *licorice*. The medication quality conforms to the national medication standard YBZ00392016, established by the State Food and Drug Administration.

All subjects accepted the recommendation of Western medicine treatment in the Diagnosis and Treatment Protocol for COVID-19 (Trial Version 7), jointly issued by the National Health Commission and the National Administration of Traditional Chinese Medicine. Antiviral therapy included treatment with oseltamivir (75 mg/tablet), PO, 1 tablet at a time, QD and arbidol (100 mg/tablet), PO, 2 tablets at a time, TID. For antimicrobial therapy, bacteriological monitoring was intensified, and antimicrobial drugs were administered promptly when there was evidence of secondary bacterial infection; penicillin, cephalosporins, floxacins, and macrolides were administered orally.

The subjects in the Jinhua group were treated with Jinhua Qinggan granules after administering Western medicine treatment: PO, 1 bag at a time, TID.

The subjects in the Western medicine group were administered Western medicine without TCM.

#### Randomization and Blinding

In this study, all patients were randomly divided into the Jinhua and Western medicine groups at a ratio of 3:1. Random numbers were generated using SAS statistical analysis software and grouped using the block randomization method. Data analysis was independently completed by professional statisticians.

#### Efficacy Evaluation and Safety Monitoring

Before and after 14 days of treatment, all subjects were assessed for symptoms, temperature, safety (blood pressure, heart rate, and respiration), medication compliance, and adverse events. The primary outcome indicators were symptom improvement rate and symptom disappearance rate after 14 days of treatment; the secondary outcome indicator was the disease aggravation rate after 14 days of treatment. During the study, all combined medications and adverse events were detected and recorded in detail in the case report table, including their occurrence, remission time, and severity.

### Statistical Analysis

Four staff members completed data entry twice independently and performed statistical analysis using SAS 9.2 software. The measurement data for normal distribution were expressed as mean ± standard deviation and subjected to an independent sample *t*-test. Count data were analyzed using the chi-square test. The continuity correction of the chi-square test was adopted for the total sample *n* ≥ 40 and theoretical number 1 ≤ T < 5. However, Fischer's exact test was used for the theoretical number T < 1 or the total sample *n* < 40. *P* < 0.05, indicating that the results were statistically significant.

## Results

### Subject Characteristics

**Figure 1:**
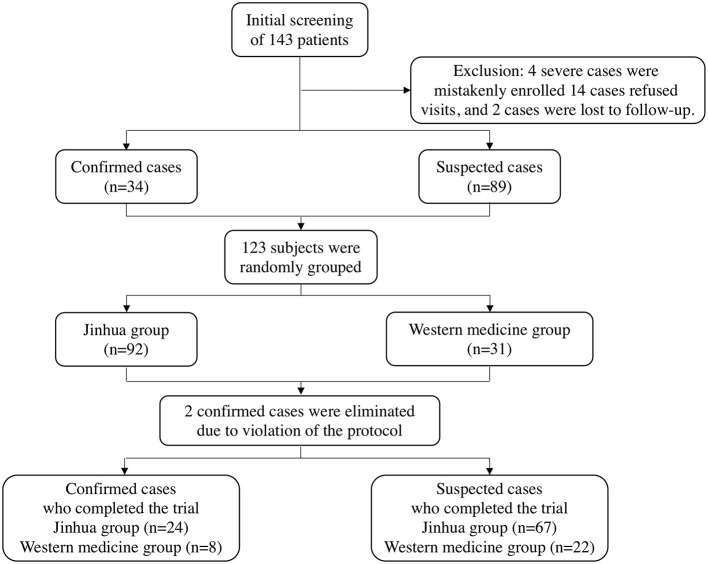
Flowchart of the screening, randomization, and treatment of subjects.

Between February 2nd to 10th, 2020, a total of 143 patients were recruited for treatment from isolation sites in Wuhan, Hubei Province. Among them, four severe cases were mistakenly enrolled, 14 patients refused visits, and two patients were lost during follow-up. As a result, 123 subjects who met the inclusion criteria were enrolled in this clinical trial (including 34 confirmed patients and 89 suspected patients). All patients were randomly divided into two groups in a 3:1 ratio; there were 92 and 31 patients in the Jinhua and Western medicine groups, respectively. At the end of the trial, two confirmed cases were eliminated due to protocol violations, and a final total of 121 patients completed the study. The patient selection process is shown in Figure 1.

Table 1 shows the baseline patient data. Patient medical history included hypertension, diabetes, hyperlipidemia, coronary artery disease, chronic obstructive pulmonary disease (COPD), and bronchial asthma. Initial symptoms included fever, fatigue, cough, and diarrhea. Except for age, but across both confirmed and suspected patients, there were no significant differences between the baseline data of the two treatment groups, including sex, height, and weight, past medical history, initial symptoms, disease diagnosis, diagnosis method, and medication before enrollment (*P* > 0.05). The average age in the Jinhua group was slightly older than that in the Western medicine group. Overall, the baseline data of the two groups were similar.

**Table 1:** Comparison of baseline data.

**Item**	**Jinhua group (*N* = 92)**	**Western medicine group (*N* = 31)**	***P*-value**
Male [% (*n*)]	45.7 (42)	48.4 (15)	0.792
Age [years, (x ± s)]	50.18 ±12.25	44.74 ±11.65	0.032
Height [cm, (x ± s)]	166.40 ±6.55	166.00 ±7.14	0.777
Weight [kg, (x ± s)]	65.218 ± 10.81	66.71 ±10.13	0.501
Smoking [% (*n*)]	17.4 (16)	9.7 (3)	0.304
Drinking [% (*n*)]	2.2(2)	9.7 (3)	0.101
Past medical history [% (*n*)]	26.1 (24)	19.4 (6)	0.450
Bronchial asthma	1.1 (1)	0.0 (0)	1.000
Coronary artery disease	1.1 (1)	0.0 (0)	1.000
Hypertension	1.1 (1)	3.2 (1)	0.442
Diabetes	20.7 (19)	12.9 (4)	0.339
Hyperlipidemia	17.4 (16)	9.7 (3)	0.304
Initial symptoms [% (*n*)]	94.6 (87)	96.8(30)	1.000
Fever	72.8 (67)	80.6 (25)	0.386
Cough	44.6 (41)	61.3 (19)	0.107
Diarrhea	10.9 (10)	12.9 (4)	0.758
Fatigue	34.8 (32)	38.7 (12)	0.693
Chest tightness and shortness of breath	15.2 (14)	6.5 (2)	0.210
Disease diagnosis [% (*n*)]			0.841
Confirmed diagnosis	27.2 (25)	29.0 (9)	-
Suspected diagnosis	72.8 (67)	71.0 (22)	-
Diagnosis method [% (*n*)]			0.581
Nucleic acid diagnosis	18.5 (17)	22.6 (7)	-
Clinical diagnosis	8.7 (8)	6.5 (2)	-

### Symptom Improvement

Table 2 presents patient, include both confirmed and suspected, symptom improvement data and Table 3 presents confirmed patient symptom improvement data. There was no significant difference in symptom improvement rates between the Western medicine and Jinhua group before and after treatment (*P* > 0.05), for both confirmed and suspected patients. Both treatment regimens improved the symptoms of fever, diarrhea, fatigue, and cough. Based on the symptom improvement rate in each group, the Jinhua group tended to have increased improvement rates for fever and poor appetite.

**Table 2:** Symptom improvement.

**Item**	**Jinhua group** **(*****N*** **=** **92)**			**Western medicine group** **(*****N*** **=** **31)**			***P*** **-value**	
	**Cases**	**Improvement Rate on Day 7[% (*n*)]**	**Improvement Rate on Day 14 [% (*n*)]**	**Cases**	**Improvement Rate on Day 7 [% (*n*)]**	**Improvement Rate on Day 14[% (*n*)]**	**Day 7**	**Day 14**
Diarrhea	23	100.0 (23)	100.0 (23)	4	75.0 (3)	100.0 (4)	1.000	-
Fever	32	100.0 (32)	96.9 (31)	10	80.0 (8)	90.0 (9)	0.052	0.424
Nausea and vomiting	26	88.5 (23)	96.2 (25)	10	90.0 (9)	100.0 (10)	1.000	1.000
Poor appetite	48	79.2 (38)	93.8 (45)	15	66.7 (10)	80.0 (12)	0.321	0.141
Sore limbs	37	73.0 (27)	91.9 (34)	11	81.8 (9)	90.9 (10)	0.552	1.000
Chest tightness and shortness of breath	47	78.7 (37)	89.4 (42)	14	78.6 (11)	92.9 (13)	0.990	1.000
Fatigue	65	69.2 (45)	86.2 (56)	19	73.7 (14)	84.2 (16)	0.709	0.831
Cough	64	59.4 (38)	73.4 (47)	24	50.0 (12)	75.0 (18)	0.429	0.882

**Table 3:** Symptom improvement of confirmed patients.

**Item**	**Jinhua group** **(*****N*** **=** **24)**			**Western medicine group** **(*****N*** **=** **8)**			***P*** **-value**	
	**Cases**	**Improvement Rate on Day 7[% (*n*)]**	**Improvement Rate on Day 14 [% (*n*)]**	**Cases**	**Improvement Rate on Day 7 [% (*n*)]**	**Improvement Rate on Day 14[% (*n*)]**	**Day 7**	**Day 14**
Diarrhea	6	83.3 (5)	100.0 (6)	0	-	-	-	-
Fever	7	100.0 (7)	85.7 (6)	3	100.0 (3)	66.7 (2)	1.000	1.000
Nausea and vomiting	7	71.4 (5)	85.7 (6)	3	100.0 (3)	100.0 (3)	1.000	1.000
Poor appetite	11	81.8 (9)	9 (81.8)	4	50.0 (2)	75.0 (3)	0.516	1.000
Sore limbs	11	9 (81.8)	9 (81.8)	3	100.0 (3)	66.7 (2)	1.000	1.000
Chest tightness and shortness of breath	11	36.4 (4)	7 (63.6)	4	75.0 (3)	75.0 (3)	0.282	1.000
Fatigue	18	61.1 (11)	72.2 (13)	4	75.0 (3)	75.0 (3)	1.000	1.000
Cough	19	52.6 (10)	73.7 (14)	5	40.0 (2)	80.0 (4)	1.000	1.000

### Medication After Treatment

Table 4 shows medication after enrollment. The confirmed and suspected patients were administered antiviral drugs and there was no significant difference in the use of antiviral drugs between the two groups (*P* > 0.05). Conversely, there was significant difference in the use of anti-infective drugs between the two groups (*P* < 0.05), and the use rate of anti-infective drugs in the Western medicine group was markedly higher than that in the Jinhua group.

**Table 4:** Medication after enrollment.

**Medication before enrollment [% (*n*)]**	**Jinhua group (*N* = 92)**	**Western medicine group (*N* = 31)**	***P*-value**
Antiviral drugs	100.0 (92)	100.0 (31)	-
Oseltamivir	87.0 (80)	100.0 (31)	0.034
Arbidol	17.4 (16)	16.1 (5)	0.872
Other	0.0 (0)	0.0 (0)	-
Anti-infective drugs	38.0 (35)	38.7 (12)	<0.001
Macrolides	0.0 (0)	100.0 (31)	-
Floxacins	28.3 (26)	25.8 (8)	-
Cephalosporins	3.3 (3)	0.0 (0)	-
Penicillin	5.4 (5)	3.2 (1)	-
Other	3.3 (3)	0.0 (0)	-

### Prognosis

Table 5 shows the duration of medication and prognosis of the confirmed patients, while the suspected patients not diagnosed as COVID-19 at last. The shortest duration of medication among confirmed patients who completed this clinical trial was four days, and the longest was 14 days. Among them, six patients developed disease aggravation, including four cases in the Jinhua group (16.7%) and 2 cases in the Western medicine group (25.0%). There was also no difference in the negative conversion rate of nucleic acid (*P* > 0.05).

**Table 5:** Duration of medication and prognosis of confirmed patients who completed the trial.

**Item**	**Jinhua group (*N* = 24)**	**Western medicine group (*N* = 8)**	***P*-value**
Duration of medication [days, (x±s)]	12.46 ± 3.54	12.13 ± 3.72	0.821
Disease aggravation [% (*n*)]	16.7 (4)	25.0 (2)	0.625
Rate difference and two-tailed 95% CI (Jinhua group–Western medicine group)	−8.333% (−41.839%, −25.173%)		
Negative conversion rate of nucleic acid [% (*n*)]	8.3 (2)	0 (0)	0.399

## Discussion

The most common symptoms of COVID-19 are fever, cough, and fatigue, as well as expectoration, headache, hemoptysis, diarrhea, and dyspnea. The principal routes of transmission of COVID-19 are via respiratory droplets or direct contact (11). The COVID-19 pathogen is a novel coronavirus named SARS-CoV-2, which is similar to the SARS pathogen and is classified as a β-coronavirus (12). Currently, there are no specific drugs for the treatment of COVID-19. Considering the advantages of TCM in treating SARS and influenza A H1N1, we selected Jinhua Qinggan granules, which have been widely applied in clinical practice, together with Western medicine, to treat COVID-19 patients with mild symptoms and examine the efficacy of TCM in the treatment of COVID-19.

Based on the symptoms of COVID-19 patients, the study team suggested that COVID-19 was a “cold and dampness epidemic” in TCM. In TCM, “epidemic” refers to plague, which is a general term for severe infectious diseases. Considering the clinical manifestations of “cold and dampness”, which include attacking the surface, blocking the lungs, and obstructing the spleen, as well as the climate of Wuhan at that time, which was overcast and rainy, it was concluded that the disease was a “cold and dampness epidemic” in TCM. Although the lungs are primarily affected by this disease, the heart and spleen are also affected. Therefore, the treatment should focus on warming “*yang*,” strengthening the body resistance, and resolving fluid, which can dispel cold, dampness, and toxic materials (13, 14). Moreover, “cold” is good at bondage, “wet” symptoms tend to linger, and a “cold and damp epidemic” can cause fever. Jinhua Qinggan granules are made from Maxingshigan Decoction and Yinqiao Powder, and have the effects of “soothing wind,” ventilating the lungs, and clearing away heat and toxic materials.

In this study, 123 patients with confirmed and suspected COVID-19 were randomly divided into the Jinhua and Western medicine groups in a ratio of 3:1. The results showed that both treatment regimens could relieve the clinical symptoms of fever, fatigue, and cough in patients with confirmed and suspected COVID-19. The rate of antibiotic use in the Jinhua group was significantly lower than that in the Western medicine group. For disease prognosis, there was no significant difference in the disease aggravation rate between the two groups after 14 days of treatment. A modern pharmacological study revealed that the main chemical components of Jinhua Qinggan granules are kaempferol, stigmasterol, and quercetin, which possess antiviral, anti-inflammatory, and immune regulatory effects (15). Using network pharmacology and high-throughput molecular docking technology, it was found that some compounds in Jinhua Qinggan granules could bind to specific target proteins of SARS-CoV-2 and inhibit its activity (16). A previous study indicated that Jinhua Qinggan granules could shorten the duration of fever, alleviate respiratory symptoms, and reduce the use of antibiotics in patients with influenza A H1N1 (17). We lowered the usage rate of antibiotics in the present study, given that Jinhua Qinggan granules have potential anti-inflammatory pharmacological effects. The honeysuckle extract in Jinhua Qinggan granules appreciably decreased the expression of IL-1, IL-6, and TNF-α proteins in the bronchoalveolar lavage fluid. Since IL-6 is one of the pivotal inflammatory factors that trigger the “cytokine storm”; the honeysuckle extract may mitigate the cytokine storm in COVID-19, reduce the risk of disease aggravation, and improve the prognosis of patients.

The present study has certain limitations. The sample size, both for total and confirmed patients, is small and there is a lack of laboratory performance indicators. At the same time, we did not record the daily changes of patients' symptoms, and it was possible that the relationship between efficacy and time may be lost. At the same time, our research is unblinded, and the collected symptoms may be affected by psychological factors. Based on the current research results, by expanding the sample size, dynamically observing clinical symptoms, and with blind methods, it is possible to find other therapeutic advantages in Jinhua Qinggan granules treatment more accurately, without the influence of psychological factors. Thus, a larger sample size and regular follow-up are needed to further clarify the clinical outcomes of patients.

## Data Availability Statement

The original contributions presented in the study are included in the article/supplementary materials, further inquiries can be directed to the corresponding author/s.

## Ethics Statement

The studies involving human participants were reviewed and approved by Ethics Committee of Hubei Provincial Hospital of TCM (HBZY2020-C01-01). The patients/participants provided their written informed consent to participate in this study. Written informed consent was obtained from the individual(s) for the publication of any potentially identifiable images or data included in this article.

## Author Contributions

FL, XL, and XT conducted the study. SL, YZha, and AF analyzed the data. XA and XX wrote the first draft of Chinese. MX, XM, and YL updated the data analysis and drafted the final manuscript. JT, JK, QZ, BL, YH, YZho, and JS revised the manuscript. All authors conceived, designed the study, read, and approved the final manuscript.

## Funding

This research was supported by the New Coronavirus Infection Pneumonia Chinese Medicine Emergency Project (2020ZYLCYJ04-1,3,4) and the Traditional Chinese Medicine Special Project for COVID-19 Emergency of National Administration of Traditional Chinese Medicine (2020ZYLCYJ04-1).

## Conflict of Interest

SL and AF were employed by GCP ClinPlus Co., Ltd. The remaining authors declare that the research was conducted in the absence of any commercial or financial relationships that could be construed as a potential conflict of interest.

## Publisher's Note

All claims expressed in this article are solely those of the authors and do not necessarily represent those of their affiliated organizations, or those of the publisher, the editors and the reviewers. Any product that may be evaluated in this article, or claim that may be made by its manufacturer, is not guaranteed or endorsed by the publisher.

## Footnotes

^1^ https://www.who.int/publications/i/item/WHO-2019-nCoV-therapeutics-2021.2

## Abbreviations

COVID-19: Coronavirus Disease 2019
TCM: Traditional Chinese Medicine
WHO: World Health Organization
SARS-CoV-2: Severe acute respiratory syndrome coronavirus
PO: peros
QD: quaque die
TID: third in die
COPD: chronic obstructive pulmonary disease.
